# Development of Multigene Expression Signature Maps at the Protein Level from Digitized Immunohistochemistry Slides

**DOI:** 10.1371/journal.pone.0033520

**Published:** 2012-03-15

**Authors:** Gregory J. Metzger, Stephen C. Dankbar, Jonathan Henriksen, Anthony E. Rizzardi, Nikolaus K. Rosener, Stephen C. Schmechel

**Affiliations:** 1 Department of Radiology, University of Minnesota, Minneapolis, Minnesota, United States of America; 2 Department of Laboratory Medicine and Pathology, University of Minnesota, Minneapolis, Minnesota, United States of America; 3 BioNet, University of Minnesota, Minneapolis, Minnesota, United States of America; Weill Cornell Medical College, United States of America

## Abstract

Molecular classification of diseases based on multigene expression signatures is increasingly used for diagnosis, prognosis, and prediction of response to therapy. Immunohistochemistry (IHC) is an optimal method for validating expression signatures obtained using high-throughput genomics techniques since IHC allows a pathologist to examine gene expression at the protein level within the context of histologically interpretable tissue sections. Additionally, validated IHC assays may be readily implemented as clinical tests since IHC is performed on routinely processed clinical tissue samples. However, methods have not been available for automated *n*-gene expression profiling at the protein level using IHC data. We have developed methods to compute expression level maps (signature maps) of multiple genes from IHC data digitized on a commercial whole slide imaging system. Areas of cancer for these expression level maps are defined by a pathologist on adjacent, co-registered H&E slides, allowing assessment of IHC statistics and heterogeneity within the diseased tissue. This novel way of representing multiple IHC assays as signature maps will allow the development of *n*-gene expression profiling databases in three dimensions throughout virtual whole organ reconstructions.

## Introduction

Disease-associated changes in gene expression patterns identified by genomics and proteomics technologies often require validation by a corroborating method for several reasons. First, discovery-phase experimental data may be confounded by specimen heterogeneity. High throughput genomics and proteomics technologies typically rely on the solubilization of tissue samples into liquid protein or nucleic acid preparations. While the assumption is often made that the samples are pure representations of a uniform disease process, each sample is actually composed of varying mixtures of diseased and non-diseased tissue constituents [1]. Further, there may be heterogeneous biomarker expression within the diseased component of a specimen, as exemplified by intratumoral heterogeneity in the expression of prognostic biomarkers in breast cancer [2], [3]. In clinical laboratories, assays that rely on tissue homogenization (sometimes referred to as “grind and bind” assays) [4] have largely been replaced by antibody-based cell staining methods that are scored in a way that accounts for heterogeneity [5]. Second, initial biomarker studies may be performed using methods not widely available, highly technically complex, or with slow test turn-around times making them clinically suboptimal [6]. Third, despite their exceptional utility in the discovery phase of experimentation, data obtained using genomics and proteomics technologies requires validation due to data quality problems beyond the issue of tissue heterogeneity, including misidentification of nucleic acid probes on gene expression microarrays [7], [8], non-specificity of probes [9], and essentially unavoidable false-positive discovery rates associated with massive multiple hypothesis testing [10]. Appropriately powered studies to validate initial results of genomics and proteomics studies often are lacking [6], [11].

Among validation methods, IHC offers important advantages for translational and clinical research. IHC is performed in the context of histologically interpretable tissue sections such that gene expression may be evaluated in specific cells (e.g., carcinoma cells versus background stromal and benign epithelial cells), or sub-cellular areas (nuclear versus cytoplasmic versus membrane) [12] at the protein level under direct microscopic visualization by a pathologist. Multiple proteins can be measured on closely spaced adjacent tissue sections, which are often cut at 4 µm thickness, or approximately one-third the diameter of a malignant cell [13], such that the relationship of multiple IHC targets can be analyzed on the same cell populations concurrently. Further, IHC is amenable to high-throughput validation of IHC targets in large numbers of patient samples utilizing tissue microarray techniques. Importantly, validated IHC assays are suitable for implementation as clinical tests since they are optimized for the standardized method of tissue handling (typically immersion of tissues in buffered formalin at specified time intervals followed by processing into paraffin blocks) that are universally applied to tissue samples in clinics, procedure rooms, radiology suites, operating rooms, and pathology laboratories [14], [15]. Indeed, it has become a norm for molecular signatures identified by gene expression profiling technologies to be implemented in clinical laboratories as multigene IHC assays, with results reportable within the rapid turn-around times expected by clinicians and patients. In the example of diffuse large B-cell lymphoma, prognostic biomarkers that distinguish tumors of germinal center versus activated B-cell subtypes were initially identified by RNA expression microarray methods [16], [17], [18], and later validated at the protein level by IHC and clinically implemented as IHC panels with results available within a one day turnaround time [19], [20], [21].

Application of a gene expression signature to patient specimens may involve the weighted summation of gene expression data to generate a positive or negative “vote” toward a relevant outcome [22], such as diseased versus non-diseased, one malignant tumor type versus another type, prognosis of aggressive versus non-aggressive clinical behavior of a tumor, and prediction of response to therapy versus non-response. The magnitude of expression of each gene comprising a signature is typically associated with a weighting factor derived from validation data that (a) is signed (positive or negative) depending on whether the gene is up-regulated or down-regulated with respect to the outcome of interest, (b) has a magnitude related to the degree to which a gene's expression is associated with the outcome of interest, and (c) may correct for differences in overall immunohistochemical staining intensity among different genes comprising the signature. A simple *n*-gene “voting” classifier within a tissue region of interest, 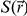, may be given as
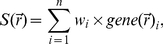
(1)where the vector 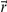 denotes the spatial dimensions of the region of interest, *w*_i_ is the weighting factor, and *gene*(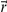)_i_ is the expression level measured for each of *n*-genes. It should be emphasized that IHC assays, because they are performed on tissue sections in which architectural features are retained, allow examination of heterogeneity of *n*-gene expression signatures across diseased tissue areas. Thus, IHC preserves a spatial aspect of data that would be lost upon tissue solubilization for standard proteomic and genomic assays.

Although studies utilizing *n*-gene classifiers indicate that IHC biomarker expression signatures hold high promise in routine clinical practice [23], there is currently a paucity of available methods to summate the weighted expression of multiple IHC markers across pathologist-annotated diseased tissue areas. In recent years, numerous manufacturers have developed whole slide imaging (WSI) systems that convert glass slides into diagnostic quality digital images that are readily accessible via network-connected computers and can provide extensive features for annotation and analysis [24]. WSI has been used in clinical practice and teaching, including primary pathologic diagnosis [25], second opinion consultation via telepathology [26], creation of teaching archives [27], and quality assurance [28]. WSI has been extensively used in research applications ranging from detailed measurement of histologic landmarks [29], [30] to quantitative IHC [31]. We have developed methods integrated with a WSI system to generate *n*-gene IHC expression profiles across tissue areas electronically annotated by a pathologist on whole-slide images. For purposes of illustration, we demonstrate the utility of these methods by generating a putative four-gene signature of prostate cancer aggressiveness in a representative prostatectomy tissue block and investigate the registration accuracy and its impact on calculated signature mapping values in 10 prostate cancer-containing blocks of prostatectomy tissue from unique subjects.

## Materials and Methods

### Genes selected

We performed IHC studies for four gene products whose expression levels are known to be related to patient outcomes in prostate cancer (PCa): *up-regulated* expression of the tumor cell proliferation marker Ki-67 (Online Mendelian Inheritance in Man [OMIM] [32] designation: MKI67), the marker of neuroendocrine tumor cell differentiation neuron-specific enolase (OMIM: ENO2), and the microvascular marker CD34 have been associated with poor prognosis [33], [34], [35], [36], [37], [38], [39], [40], whereas *down-regulated* expression of prostate specific acid phosphatase (PSAP; OMIM: ACPP) is associated with higher tumor grade [41] and unfavorable pathologic features in prostatectomy specimens that follow diagnoses of PCa on transurethral resections [42]. It should be stressed that these markers were chosen simply to illustrate the developed methods and because of their availability for the automated immunostainer; we do not mean to suggest that the expression signature of these gene products constitutes a validated prognostic signature.

### IHC assays

After obtaining written consent from research subjects and approval from the University of Minnesota Institutional Review Board, unstained adjacent 4 µm sections of formalin-fixed paraffin-embedded prostate tissue were cut from 10 prostatectomy blocks representing 10 unique subjects and PCa of different histologic grades. One section was stained with hematoxylin and eosin (H&E) and, using an automated immunostainer (Ventana Medical Systems, Tucson, AZ), adjacent sections were stained with primary antibodies, washed, and then a brown precipitate was developed at sites of primary antibody binding through use of a peroxidase-conjugated second step antibody and a 3,3-diaminobenzidine (DAB) reagent (Bond Polymer, Leica, Richmond, IL). For all ten cases, primary antibodies directed against the protein products of *MKI67* (antibody clone MM1, Leica Microsystems, Bannockburn, IL), *CD34* (QBend/10, Ventana) and *ACPP* (clone PASE/4LT, Cell Marque, Rocklin, CA) were used as they represent a wide range of staining intensities from relatively low to medium to high, respectively. In one additional case, used to demonstrate the creation of a four-gene signature map, a fourth IHC slide was generated with primary antibodies directed against the protein product of *ENO2* (clone BBS/NC/V1-h14, Covance, Princeton, NJ). IHC slides, and a final negative control section in which primary antibody incubation was omitted, were counterstained with hematoxylin. All slides were cover-slipped. The negative control sections were visually inspected by the study pathologist (SCS) who validated the absence of DAB signal.

### Whole Slide Imaging

Slides were scanned at 20× magnification (0.5×0.5 µm^2^ pixel resolution) using a WSI instrument (ScanScope CS, Aperio, Vista, CA) fitted with a 20×/0.75 Plan Apo objective lens (Olympus, Center Valley, PA). Images were saved in SVS format (Aperio) which is essentially a TIFF compressed with JPG2000. Images were saved on a server equipped with server software (ImageServer, Aperio) and retrieved using file management software (Spectrum, Aperio). Pathologist-annotated tumor regions were drawn using a pen tablet screen (Cintiq 21UX, Wacom, Kazo-shi, Saitama, Japan) on whole slide images viewed at high resolution using the Aperio system's annotation software (ImageScope 10, Aperio). Regions of cancer were separately labeled with their Gleason Grade (e.g. 3+3, 3+4, etc.) within different virtual planes (“layers”) of the reference slide image file.

### Generating Signature Maps

**Figure 1:**
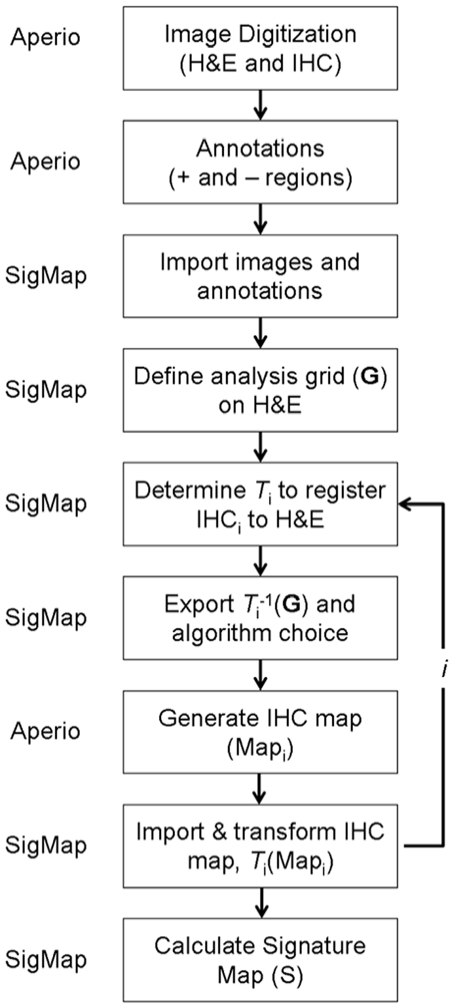
The workflow for generating signature maps. The steps to generate a signature map take place on the host computer running the interface software (labeled as SigMap) and on Aperio's ImageServer (labeled as Aperio). The location at which each step is performed is defined to the left of each step. After digitizing both the reference H&E and IHC data on the Aperio system, the data are downloaded to SigMap. This data may include pathologist annotations (i.e. capsule and graded areas of cancer in the case of the prostate). A user defined grid (**G**) is then defined in SigMap on the coordinate space of the reference (H&E) data. The inverse of the transformation (*T*_i_^−1^) required to register each IHC image (IHC_i_) to the H&E is used to match **G** to the full resolution IHC_i_ data as it exists on Aperio's ImageServer. The transformed grid, *T*_i_^−1^ (**G**), defines regions from which IHC values are determined (using the pre-selected algorithm) and combined to form IHC maps. The transformation of the IHC maps after downloading to SigMap, *T*_i_(Map_i_), places each in the coordinate space of the reference image allowing them to be combined into a signature map (S) using Equation 1.

**Figure 2:**
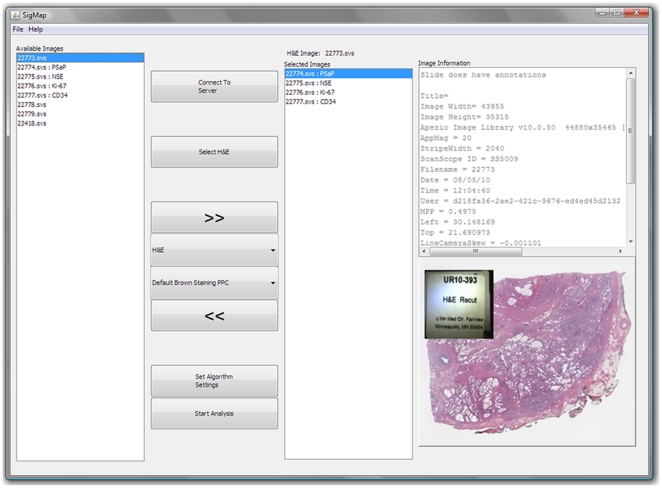
The main SigMap program window. Within the main program window the user selects the reference H&E image (designated “Select H&E”), the IHC images, and the analysis macro to be used. The Aperio Positive Pixel Count algorithm was employed in this example using default threshold settings (designated as “Default Brown Staining PPC”). If desired, threshold settings may be adjusted by navigating to the algorithm settings menu by selecting “Set Algorithm Settings”.

A software interface, which will be referred to as SigMap, was written in the Java programming language [43] to generate IHC signature maps (see Table 1 for glossary of terms) through a multistep process described below and detailed in Figure 1. Upon launching SigMap, existing WSI thumbnails and annotations in an XML format were downloaded. From the list of available images, the user selected the reference image (an H&E-stained slide image in this example), the IHC slides to be analyzed, and the analysis algorithm macro to be used for the antibody markers of interest (Figure 2). The default algorithm, Positive Pixel Count (v9, Aperio), was configured to detect the fraction of pixels that exceed pre-set (user-adjustable if desired) weak, moderate, and strong threshold limits in the brown colorimetric channel. If desired, other analysis macros available to the user within Spectrum could be selected from a drop-down menu. If present for a particular reference slide, pathologist annotations were also downloaded for subsequent sub-region analysis. Communication with ImageServer for downloading annotations and imaging data is accomplished using an HTTP GET/POST protocol provided by Aperio [44] which also allows SigMap to initiate slide analysis using pre-existing algorithms (Algorithm Framework, Aperio).

**Table 1:** Glossary of Terms.

Reference slide	Digitized slide on which annotations are drawn
IHC slide	Digitized IHC-stained slide (adjacent section relative to reference slide)
IHC score	Quantified IHC data at a single grid region of an image
IHC map	2D representation of IHC scores across an IHC slide
IHC signature score	Multiple IHC scores summated to represent quantified IHC data at a single grid region of an image
IHC signature map	Multiple IHC scores representing *n*-gene protein expression across the annotation area of the reference slide

**Figure 3:**
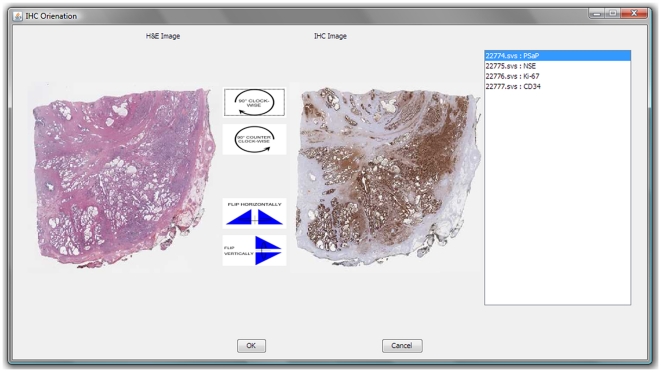
SigMap's registration window. Shown is the registration window in which the user performs rough alignment of each IHC slide (the section stained with antibody directed against ACPP illustrated to the right) to the reference slide (H&E stained section illustrated to the left). Manipulations may include clockwise or counterclockwise rotations in 90 degree increments, or vertical or horizontal flipping to achieve rough image alignment prior to launching TurboReg which is done by selecting “OK”.

**Figure 4:**
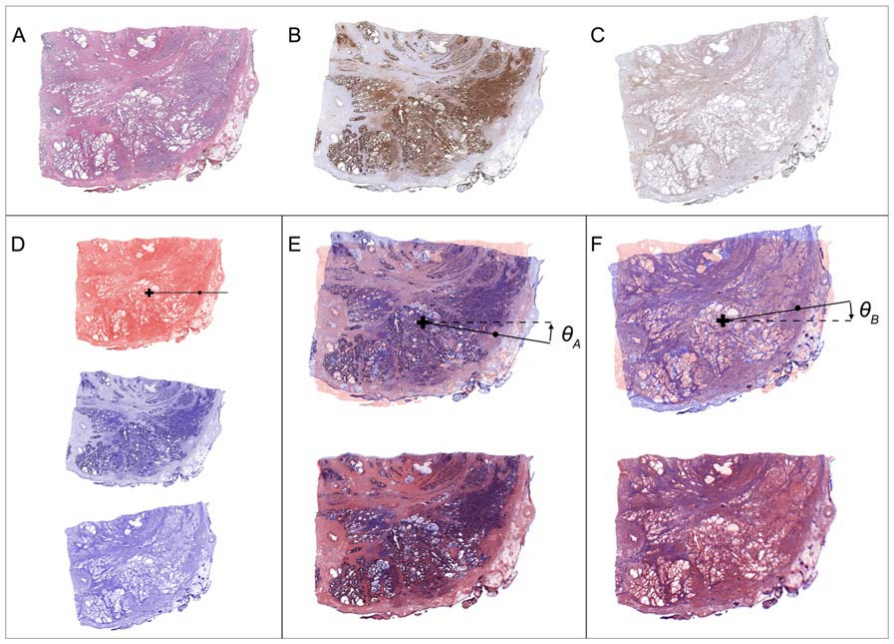
Alignment results using TurboReg. (**A**) Image of H&E-stained reference section image. (**B**) Image of section stained for ACPP previously brought into rough alignment with reference image by user. (**C**) Image of section stained for ENO2 previously brought into rough alignment with reference image by user. (**D**) Monochromatic images of A–C generated for purposes of overlay illustrations. (**E**) TurboReg translated and rotated (angle ⊖_A_) the image of ACPP-stained section until a mean location error was minimized. (**F**) Similarly, TurboReg translated and rotated (angle ⊖_B_) the image of ENO2-stained section to minimize mean location error between this image and the reference H&E-stained image. The final registered IHC image is overlaid on the reference H&E image in the bottom of panels (**E**) and (**F**). Values for translations and rotation determined by TurboReg along with the initial coarse registration steps were stored in the software for later use in transforming grid locations from the reference to the IHC images when generating IHC and signature maps.

After downloading, the whole-slide IHC images were each registered to the reference image (the H&E stained slide image in this example) through a two-step process involving coarse manual alignment followed by an automatic fine registration using a software module called TurboReg [45], [46]. During the first step in image alignment, each IHC slide was brought into rough alignment with the reference slide. Visually guided manipulations were used to flip and rotate each IHC slide so that it was in rough alignment with the reference image (Figure 3). TurboReg software then automatically completed the registration process by minimizing the mean location error through rigid body transformations (i.e. translations and rotations) (Figure 4).

**Figure 5:**
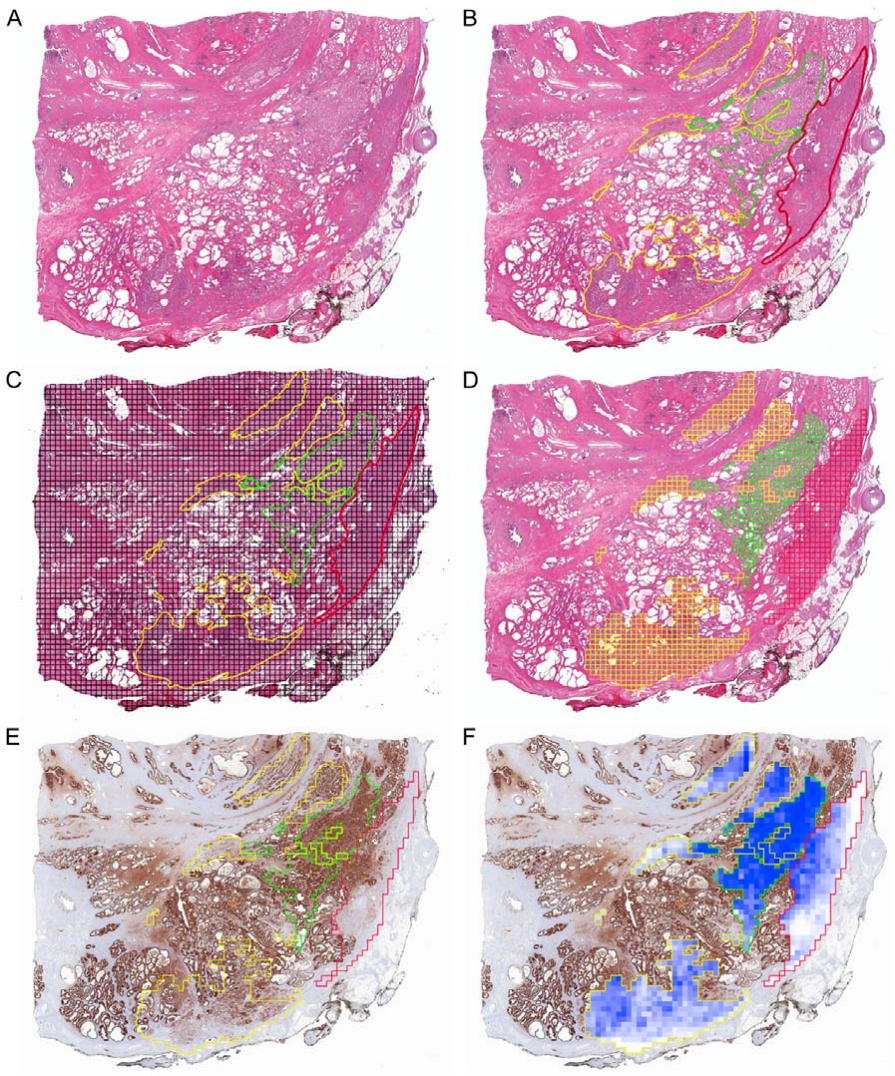
Generation of an IHC Map. (**A**) Reference H&E-stained section. (**B**) Pathologist annotations drawn in XML markup format using a pen tablet screen. In this example, prostate cancer of Gleason sum score 3+3 is outlined in green, score 3+4 is outlined in yellow and 4+3 is outlined in red. (**C**) IHC Map overlaid the annotated image with a grid with dimensions 0.25×0.25 mm^2^. (**D**) Grid squares that were within pathologist annotated areas were retained. (**E**) Contours of the annotated regions at the resolution of the analysis grid registered to the ACPP image. (**F**) The intensity of ACPP staining within each grid location in (F) was determined by using the results of the Positive Pixel Count algorithm (default settings) multiplied by the assigned weighting factor. The weighted value for each grid square was termed an IHC score. The two-dimensional depiction of IHC Score values was termed an IHC Map.

After registration, a virtual grid of user-defined resolution was made on the reference image using SigMap (Figure 5). In this example, a grid with a resolution of 0.25 by 0.25 mm^2^ was used. Grid locations outside of the tissue boundary were discarded, and SigMap was further set to discard grid locations outside of annotation regions as determined by the Monte Carlo method [47], in which random points (an adjustable number, by default 500 points) within each grid location were generated and tested for whether they resided within an annotated area. If a threshold (an adjustable percentage, by default 50%) of these points were within the annotated area, the grid location was retained, and if not, the grid location was discarded. Adjustment to require inclusion of more points would lower the threshold for discarding grid locations, and thus only retain grid locations further interior to annotations. Additionally, the pathologist could review the IHC slides and mark (using a “negative pen tool” function in ImageScope) areas of any slide image (reference or IHC stained) that lacked diagnostic tissue, contained artifact, etc.; SigMap would remove these negatively selected regions from the analysis for all stains.

Using the inverse of the previously saved transformations determined from the image alignment process, the retained grid locations generated on the reference H&E image were transformed to the native orientation of each full resolution IHC stained image. The transformed grid locations were written to a file in Aperio's annotation XML format and attached to each IHC slide by uploading to ImageServer. At each grid location, the intensity of each IHC stain was computed (termed an IHC score) using Positive Pixel Count as the selected analysis algorithm. The IHC scores across an entire grid were used to generate IHC maps after transforming back to match the reference H&E orientation.

Using gene-specific weighting values, weighted IHC scores for the *n*-genes were summated across all IHC stains at the same grid location (termed an IHC signature score) using Equation 1. In this study, the magnitude of the weights for MKI67, EN02, CD34 and ACPP were calculated to normalize the mean IHC score for each stain from the regions of annotated cancer across all subjects, and the sign of each weight reflected whether published studies cited above suggest that these proteins are up- or down-regulated in aggressive (relative to non-aggressive) PCa. IHC signature scores were displayed as a two dimensional representation oriented to the reference tissue section (termed an IHC signature map). After completing this process, output files were recorded into a specified folder, the grid created by SigMap was removed and the original pathologist annotations were restored on ImageServer.

When generating the IHC maps and IHC signature maps, the process alternated between Aperio and SigMap software environments. Processes which required the full resolution data (0.5×0.5 µm^2^), such as defining regions of disease and algorithm analysis, were performed on Aperio, whereas downsampled data were used within SigMap for registration, grid definition and signature map generation. There were two reasons to separate the tasks in this manner. First, performing image manipulations in the WSI system would be highly computationally intensive, since the uncompressed images averaged 5.4 GB for the ten cases in this study whereas the size of the 0.5× images used in SigMap were an average size of 3.4 MB (20×20 µm^2^). Second, the functionality to register digitized slides and create signature maps did not exist on WSI systems. Thus SigMap performed manipulations on the lower resolution data in SigMap and imposed manipulations on the high resolution source data via transformed analysis grids applied in the Aperio system.

### Assessment of Registration Performance

In order to evaluate the performance of the signature mapping software and the effect of registration errors on IHC map and IHC signature map statistics, gold standard registrations were performed using Photoshop (version CS5 Extended, Adobe, San Jose, CA). This optimal registration was accomplished by visually matching each IHC image to its reference H&E image with particular attention to the regions of annotated cancer. These pre-registered data were uploaded onto the Aperio server and signature mapping was performed as described above but without any additional registration steps. These data are referred to as the benchmark data and, for the purposes of this study, represent the best registration achievable. The same cases were also processed with SigMap with the slides in their native scanned orientations, allowing the user and TurboReg to register the digitized IHC to the reference H&E data as described in the methods section (referred to as the native data).

Three IHC slides for each of 10 cases were used to assess registration performance and the impact on generated IHC Signature values over regions of annotated cancer. To quantify the registration accuracy, five landmarks, distributed across the image, were placed on each native IHC image and at corresponding spatial locations on the reference H&E based on prevalent anatomic features. The error was assessed for each IHC image by calculating the vector distance between the reference H&E and native IHC image in all 150 resulting pairs of landmarks using standard tools in Photoshop. The vector lengths between the reference and native landmarks were recorded as the registration error (in micrometers).

The generated IHC signature scores were produced for each annotated region of cancer. Box plots indicating minimum, first quartile, median, third quartile and maximum values of the signature scores from each annotated cancer region were used to display IHC signature score data for both the benchmark and the native data.

## Results

### Signature Map Generation

**Figure 6:**
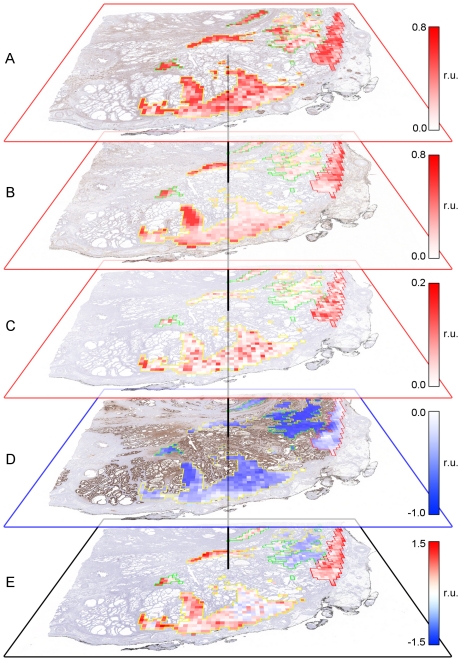
Generation of an IHC Signature Map. Displayed values of the IHC maps and IHC signature maps are in relative units (r. u.). (**A**) IHC Map for ENO2, shown in red since the weighting factor was positively signed (higher expression is associated with aggressive disease) and thus higher expression was shown as more intense red. (**B**) IHC Map for CD34 (also shown in red due to positively signed weighting factor). (**C**) IHC Map for MKI67 (also shown in red due to positively signed weighing factor). (**D**) IHC Map for ACPP, shown in blue since the weighting factor was negatively signed (higher expression is associated with non-aggressive disease) and thus higher expression was shown as more intense blue. (**E**) The weighted sum of IHC Scores (termed an IHC Signature Map) were projected in grid squares across annotated tumor areas of Gleason scores 3+3, 3+4, and 4+3 outlined in green, yellow and red, respectively.

**Figure 7:**
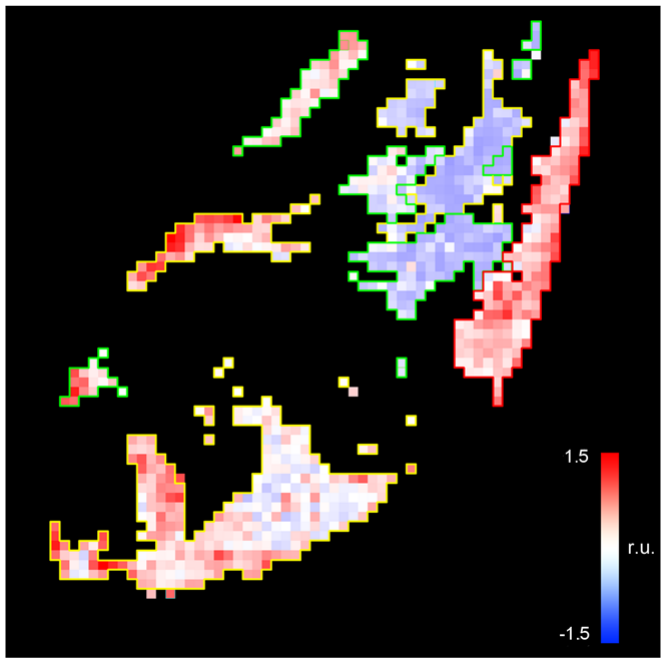
Four-gene IHC signature map. The IHC signature map is displayed in relative units (r. u.), projected against a black background to illustrate the four-gene signature map in each pathologist-annotated area (tumor areas of Gleason sum scores 3+3, 3+4, and 4+3 outlined in green, yellow and red, respectively).

The IHC scores across the entire grid shown in Figure 5 were used to generate IHC maps for all four genes after transforming back to match the reference H&E orientation (Figure 6). IHC signature scores were calculated using Equation 1 across all grid locations and the results of all processed grid locations were displayed as an IHC signature map (Figures 6 and 7). The weights used for generating the signature map were −1.0, 19, 4 and 4 for ACPP, CD34, MKI67 and ENOS, respectively. The weights for ACPP, CD34 and MKI67 were generated using all annotated cases while the weight for ENOS was derived from the single case for which that stain was performed.

The approximate time to generate a signature map depends on the number of IHC slides included, the complexity of the required annotations and the area over which IHC scores are to be generated. The total time to generate the IHC signature map shown in Figure 7 was approximately 64 minutes: 35 minutes to digitize 5 slides; 4 minutes to annotate the reference H&E; 2 minutes to connect to the server, select slides, stains and analysis algorithms in SigMap; 1 minute for registration and grid generation; 20 minutes to analyze of for all 4 IHC slides (5 minutes per slide); and 1 minute for generating final results.

### Registration Analysis

**Figure 8:**
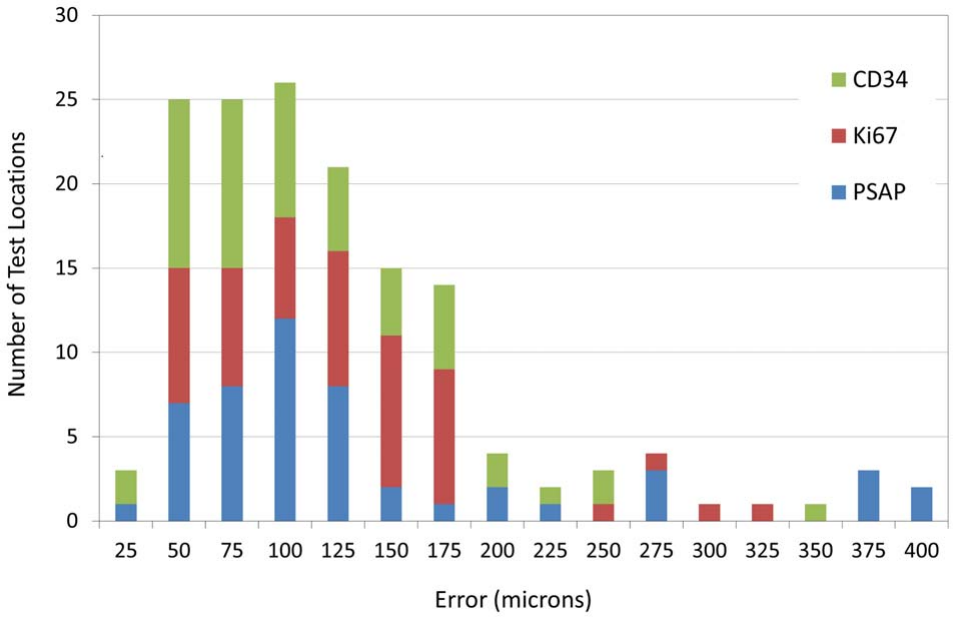
Registration errors between the IHC and H&E images. Registration errors between each of the 3 IHC datasets registered to the reference H&E data for all 10 subjects. The error estimate is based on the vector distance between landmarks placed on the same anatomic features present in the registered IHC and reference H&E slides. The histogram contains 5 measurements for each of 3 IHC datasets for 10 cases resulting in a total of 150 error estimates. The histogram shows the stain dependent errors for ACPP (blue), MKI67 (red) and CD34 (green).

The error calculated for all 150 landmark pairs, color coded by protein, is shown in the histogram in Figure 8. Stains had similar skewed error distributions with (median µm, maximum µm) values of (114, 317), (88, 350), and (94, 398) for MKI67 (low intensity stain), (CD34 medium intensity staining) and ACPP (high intensity staining), respectively. Median errors of 88–114 µm correspond to 6–7 cell diameters, and the maximal error of 398 µm corresponds to 27 cell diameters, assuming a malignant PCa cell diameter of 15 µm [13]. There was no apparent relationship between staining intensity and error in registering IHC images with reference H&E images.

### Effect of Registration Method on Signature Map Data

**Figure 9:**
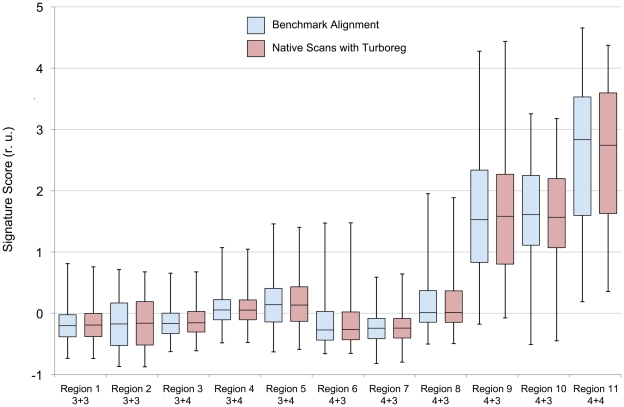
IHC signature scores from 11 annotated regions of cancer from 10 subjects. The benchmark (blue) and native (red) data for the same region are shown immediately adjacent to each other for comparison. For each region, the minimum, 1^st^ quartile, median, 3^rd^ quartile and the maximum values are shown. The data are sorted first by grade and then by the median of the benchmark data within each grade.

To evaluate the effect of registration method on IHC signature maps, IHC signature maps were generated for 10 cases using a benchmark method and compared with IHC signature maps generated using the SigMap method of initial manual course registration followed by TurboReg fine registration. Data for IHC signature scores within 11 annotated cancer regions were available for these ten cases (one case had two distinct areas of cancer in the reference H&E image), and are presented in Figure 9 as box plots for the benchmark (blue) and the native (red) IHC signature values from these regions. The signature scores from the native data, which underwent the registration procedure of SigMap, closely match the optimally registered results from the benchmark data. Therefore, registration error associated with manual course registration followed by TurboReg fine registration appears to have essentially no impact on signature scores within annotated regions of cancer.

## Discussion

Methods to generate maps of *n*-gene biomarker signatures within pathologist-annotated tissue regions were developed by combining the functionality of a Java software interface, SigMap, with that of a commercial digitization platform, Aperio. By itself, the Aperio system provides diagnostic quality digitization, annotation tools and analysis functionality, but there was no way to combine multiple IHC and H&E data sets. Signature maps, as described in this paper, provide a unique insight into the spatial distribution of gene signatures which has the potential to extend the research and diagnostic potential of immunohistochemistry studies.

### Factors Affecting Signature Map Generation

There are several factors which can potentially impact the signature scores generated by SigMap including the weights used for combining the multiple IHC datasets, consistency of IHC staining and the analysis algorithm used for generating the IHC score, variability in annotating cancer regions and the accuracy of the registration procedure. In this study, while the magnitude of the weighting factors were simply used to normalize the mean expression level of each IHC marker, the polarity of the weights reflected published data regarding whether each protein would be expected to be up- or down-regulated in aggressive (versus non-aggressive) prostate cancer. In clinical application, the sign and magnitude of each weighting factor would be determined through a validation study correlating expression levels with an outcome variable, and then weighting factors would be applied uniformly across all patient samples to allow cross-case comparisons.

Staining consistency, stain quantification and annotation variability may also impact the reliability of IHC signature scores. To minimize the potential impact of variable staining and quantification, an automated immunostainer and antibodies optimized for use on that platform were used in this study. However, in general, staining consistency and quantification algorithms would need to be validated before clinical use. While not investigated in the current study, it is expected that between pathologists, the margins of annotated regions of cancer may vary. The potential impact of this variability on summary statistics for annotated regions requires subsequent study.

### Registration Methods

The effect of registration on the generation of signature scores was explicitly tested in this study. The rigid registration strategy used in the current implementation of SigMap is very basic. This approach minimizes the mean-square difference between the reference H&E and the IHC images using translations and rotations. Using the mean-square difference to optimize the registration process assumes that the spatial distributions and signal intensities between the images are similar for best results. Although the digitized slides are converted to greyscale before registration, the distribution of signal intensities can vary widely between the H&E and various IHC datasets. Despite this fact, this registration method performed well as the overall anatomic structure apparently dominates the registration yielding excellent results. This can be appreciated by the median registration errors of 99 µm as determined from the control points between the benchmark and the native datasets. This registration error is smaller than the grid resolution of 250 µm used to generate the presented signature maps, which likely explains the negligible impact on the generated IHC scores, and IHC signature scores reported in Figure 9.

The image alignment method could be further improved by automating the process and by potentially implementing deformable registration methods to address tissue compression and stretching which occurs when making slides. For example, Cooper et al. have demonstrated an automatic non-rigid, feature-based strategy to register histological sections with different stain types [48]. However, it is unclear how such methods would perform when large rotational differences are present or when IHC sections are flipped with respect to the reference H&E.

While alternative registration strategies are possible within SigMap, there are some limitations as to what can be realized on the Aperio system. Each grid location on Aperio must be defined by a series of straight edges which connect at vertices. Therefore, if deformable registration strategies are used to match the IHC data to the reference H&E, the subsequent grid made from these transformations could only be approximated. In general, these transformations should also be invertible.

### Processing time

Most of the total time required for generating signature maps is spent on processes which run in the background. Out of the 64 minutes it took to generate the signature map in Figure 7, 35 minutes were spent scanning the H&E and IHC slides and 20 minutes on analysis which is performed on the ImageServer. The amount of pathologist time is minimal and limited to the time required to annotate the digitized H&E (approximately 4 minutes per slide), while the other laboratory staff time to setup slides for scanning and run SigMap is minimal (approximately 10 minutes). Newer WSI imaging platforms are several-fold faster than the study instrument. Further, the analysis time on the ImageServer could be greatly reduced by either fully utilizing multi-core processing or GPU acceleration since the process to analyze the IHC score within each grid location is highly parallelizable.

### Potential Role of IHC Signature Maps

*N*-gene expression profiling has broad applicability in anatomic pathology as new diagnostic, prognostic, and predictive protein biomarker panels are developed to individualize approaches to patient care. Compared to single gene models, multigene expression profiles of cancer and other complex diseases may more accurately classify disease, prognosticate clinical outcome, and predict response to therapy. In prostate cancer, numerous publications have identified *n*-gene molecular signatures that are correlated with biochemical failure versus non-failure following prostatectomy [49], [50], [51], [52], [53], [54].

Validation of *n*-gene expression signatures may be best performed using IHC. Several recent papers suggest that *n*-gene expression profiling assessed at the protein level by IHC are amenable to large-scale, tissue microarray-based validation studies, which yield similar tumor sub-typing as multigene signatures assessed by molecular (qRT-PCR or microarray) methods [55], [56]. IHC has been used to validate the association of gene expression profiles in prediction of cancer patient response to treatment [57], and prognosticating risk of metastasis [23], [58] and other outcome measures [59]. Once validated, IHC-based assays may be rapidly deployed as clinical tests [19], [20], [21] on standardly fixed and processed tissue sections, with rapid (typically same-day) turn-around times optimal for clinical practice. Formalin fixation and paraffin embedding of tumor tissues have been recently reinforced by the College of American Pathologists and the American Society of Clinical Oncology as being reliable for generating reproducible intra- and inter-laboratory measurements of gene expression levels [14], [15].

Alternatives to using signature maps for multigene signature analysis include laser capture microdissection of tissue sub-regions followed by solubilization of captured tissues for multigene molecular analysis [60]. However, the signature maps constructed by SigMap much more directly addresses gene expression in tissue sub-regions without need for tissue microdissection and allow the assessment of expression heterogeneity. Another alternative is immunofluorescence, which allows staining with multiple antibodies, each labeled with a distinct fluorescent marker [61], [62]. Signature maps are superior to immunofluorescence results for two reasons. Routine colorimetric IHC with hematoxylin counterstaining allows bright-field determination of tissue architecture under pathologist direct visualization that is superior to dark field methods used in immunofluorescence [63], [64]. Further, whereas special optimization procedures often needed to multiplex fluorescently-labeled antibody assays, SigMap allows signature determination from multiple, routine, single antibody staining assays.

The benefits of evaluating biomarker expression under direct microscopic visualization in the context of histologically interpretable tissue sections, rather than “grind and bind” methods such as those used in most biochemical experiments including most genomics and proteomics methods, is perhaps best illustrated by the history of estrogen receptor (ER) quantitation in breast cancer. Although ER quantification was initially performed by solubilizing snap-frozen tumor tissue and then using tissue extracts in biochemical ligand binding assays (LBA), IHC assays rapidly replaced biochemical assays. IHC allows the pathologist to restrict analysis only to visualized tumor cells (small ER-positive tumors may otherwise yield false-negative LBA results due to damping of signal by abundant benign background tissue elements), is less expensive, is amenable to standardized tissue handling methods, and allows direct correlation with other molecular markers assayed on adjacent sections (reviewed in references [65], [66], [67]).

Assessment of the degree of heterogeneity of single-gene or multigene signature expression within diseased tissue areas is an additional strength of signature maps. Heterogeneity in *HER-2* amplification occurs in 5–30% of breast cancer tumors [68] and there is also considerable spatial heterogeneity in HER-2 protein expression, particularly among cases with equivocal (2+) overall staining [69]. There is data to suggest that this heterogeneity may be clinically important: 2–5% of women whose primary breast tumors lacked definite *HER-2* amplification or HER-2 protein (3+) over-expression nevertheless had lymph node metastases that were amplified/over-expressed [70], suggesting that small HER-2-positive tumor subclones present in the primary tumor may evolve to give rise to metastatic tumor growths. Signature maps will allow the degree of spatial heterogeneity of single-gene expression and multigene signatures across large tumor areas present on pathologist annotated whole slides to be quantitatively assessed. As such, these *n*-gene expression maps may be useful in studies evaluating the potential importance of heterogeneity in patient prognostic and predictive assays.

Finally, signature maps may be useful to develop multigene expression signatures throughout virtually reconstructed whole organs as they retain their spatial relationships to the H&E-stained reference whole slide images. Using the developed methods, transformations (flips, translations and rotations), needed to assemble individual images into reconstructed whole organs, may be mathematically applied to IHC signature maps in a manner similar to the way such transformations are handled by SigMap, such that three-dimensional multigene expression signatures may be displayed in the spatial context of reconstructed organ histology. Spatially co-registering virtual whole organ reconstructions with preoperative *in vivo* anatomic and functional imaging methods such as computed tomography (CT), positron emission tomography (PET) and magnetic resonance imaging (MRI) would then allow direct comparison in three dimensions between features obtained by imaging and features including multigene signatures obtained by pathologic evaluation. For example, genes whose expression patterns are validated to be highly significant for predicting disease aggressiveness could be co-registered with imaging and used as a gold standard for identifying imaging biomarkers that assess disease aggressiveness *in vivo*. This would expand upon previous work in mouse models in which spatially mapped gene expression data was overlaid on detailed anatomic information [71], [72].

## References

[1] Shukla CJ, Pennington CJ, Riddick AC, Sethia KK, Ball RY (2008). Laser-capture microdissection in prostate cancer research: establishment and validation of a powerful tool for the assessment of tumour-stroma interactions.. BJU Int.

[2] Nassar A, Radhakrishnan A, Cabrero IA, Cotsonis GA, Cohen C (2010). Intratumoral heterogeneity of immunohistochemical marker expression in breast carcinoma: a tissue microarray-based study.. Appl Immunohistochem Mol Morphol.

[3] Hanna W, Nofech-Mozes S, Kahn HJ (2007). Intratumoral heterogeneity of HER2/neu in breast cancer–a rare event.. Breast J.

[4] Cummings M, Iremonger J, Green CA, Shaaban AM, Speirs V (2009). Gene expression of ERbeta isoforms in laser microdissected human breast cancers: implications for gene expression analyses.. Cell Oncol.

[5] Harvey JM, Clark GM, Osborne CK, Allred DC (1999). Estrogen receptor status by immunohistochemistry is superior to the ligand-binding assay for predicting response to adjuvant endocrine therapy in breast cancer.. J Clin Oncol.

[6] McShane LM, Altman DG, Sauerbrei W, Taube SE, Gion M (2005). Reporting recommendations for tumor marker prognostic studies (REMARK).. J Natl Cancer Inst.

[7] Schmechel SC, LeVasseur RJ, Yang KH, Koehler KM, Kussick SJ (2004). Identification of genes whose expression patterns differ in benign lymphoid tissue and follicular, mantle cell, and small lymphocytic lymphoma.. Leukemia.

[8] Tu IP, Schaner M, Diehn M, Sikic BI, Brown PO (2004). A method for detecting and correcting feature misidentification on expression microarrays.. BMC Genomics.

[9] Kapur K, Jiang H, Xing Y, Wong WH (2008). Cross-hybridization modeling on Affymetrix exon arrays.. Bioinformatics.

[10] Norris AW, Kahn CR (2006). Analysis of gene expression in pathophysiological states: balancing false discovery and false negative rates.. Proc Natl Acad Sci U S A.

[11] Freedman AN, Seminara D, Gail MH, Hartge P, Colditz GA (2005). Cancer risk prediction models: a workshop on development, evaluation, and application.. J Natl Cancer Inst.

[12] Rexhepaj E, Brennan DJ, Holloway P, Kay EW, McCann AH (2008). Novel image analysis approach for quantifying expression of nuclear proteins assessed by immunohistochemistry: application to measurement of oestrogen and progesterone receptor levels in breast cancer.. Breast Cancer Res.

[13] Stott SL, Hsu CH, Tsukrov DI, Yu M, Miyamoto DT (2010). Isolation of circulating tumor cells using a microvortex-generating herringbone-chip.. Proc Natl Acad Sci U S A.

[14] Wolff AC, Hammond ME, Schwartz JN, Hagerty KL, Allred DC (2007). American Society of Clinical Oncology/College of American Pathologists guideline recommendations for human epidermal growth factor receptor 2 testing in breast cancer.. Arch Pathol Lab Med.

[15] Hammond ME, Hayes DF, Dowsett M, Allred DC, Hagerty KL (2011). American Society of Clinical Oncology/College of American Pathologists guideline recommendations for immunohistochemical testing of estrogen and progesterone receptors in breast cancer.. Arch Pathol Lab Med.

[16] Alizadeh AA, Eisen MB, Davis RE, Ma C, Lossos IS (2000). Distinct types of diffuse large B-cell lymphoma identified by gene expression profiling.. Nature.

[17] Shipp MA, Ross KN, Tamayo P, Weng AP, Kutok JL (2002). Diffuse large B-cell lymphoma outcome prediction by gene-expression profiling and supervised machine learning.. Nat Med.

[18] Poulsen CB, Borup R, Nielsen FC, Borregaard N, Hansen M (2005). Microarray-based classification of diffuse large B-cell lymphoma.. Eur J Haematol.

[19] Chang CC, McClintock S, Cleveland RP, Trzpuc T, Vesole DH (2004). Immunohistochemical expression patterns of germinal center and activation B-cell markers correlate with prognosis in diffuse large B-cell lymphoma.. Am J Surg Pathol.

[20] Muris JJ, Meijer CJ, Vos W, van Krieken JH, Jiwa NM (2006). Immunohistochemical profiling based on Bcl-2, CD10 and MUM1 expression improves risk stratification in patients with primary nodal diffuse large B cell lymphoma.. J Pathol.

[21] Adams H, Tzankov A, d'Hondt S, Jundt G, Dirnhofer S (2008). Primary diffuse large B-cell lymphomas of the bone: prognostic relevance of protein expression and clinical factors.. Hum Pathol.

[22] Golub TR, Slonim DK, Tamayo P, Huard C, Gaasenbeek M (1999). Molecular classification of cancer: class discovery and class prediction by gene expression monitoring.. Science.

[23] Giusiano S, Secq V, Carcopino X, Carpentier S, Andrac L (2010). Immunohistochemical profiling of node negative breast carcinomas allows prediction of metastatic risk.. Int J Oncol.

[24] Yagi Y, Gilbertson JR (2008). A relationship between slide quality and image quality in whole slide imaging (WSI).. Diagn Pathol.

[25] Gilbertson JR, Ho J, Anthony L, Jukic DM, Yagi Y (2006). Primary histologic diagnosis using automated whole slide imaging: a validation study.. BMC Clin Pathol.

[26] Wilbur DC, Madi K, Colvin RB, Duncan LM, Faquin WC (2009). Whole-slide imaging digital pathology as a platform for teleconsultation: a pilot study using paired subspecialist correlations.. Arch Pathol Lab Med.

[27] Li L, Dangott BJ, Parwani AV (2010). Development and use of a genitourinary pathology digital teaching set for trainee education.. J Pathol Inform.

[28] Ho J, Parwani AV, Jukic DM, Yagi Y, Anthony L (2006). Use of whole slide imaging in surgical pathology quality assurance: design and pilot validation studies.. Hum Pathol.

[29] Pedro RN, Thekke-Adiyat T, Goel R, Shenoi M, Slaton J (2010). Use of tumor necrosis factor-alpha-coated gold nanoparticles to enhance radiofrequency ablation in a translational model of renal tumors.. Urology.

[30] Jiang J, Goel R, Schmechel S, Vercellotti G, Forster C (2010). Pre-conditioning cryosurgery: Cellular and molecular mechanisms and dynamics of TNF-alpha enhanced cryotherapy in an in vivo prostate cancer model system.. Cryobiology.

[31] van Niekerk CG, van der Laak JA, Borger ME, Huisman HJ, Witjes JA (2009). Computerized whole slide quantification shows increased microvascular density in pT2 prostate cancer as compared to normal prostate tissue.. Prostate.

[32] Online Mendelian Inheritance in Man OTM-NIoGM website, Johns Hopkins University (Baltimore, MD) and National Center for Biotechnology Information, National Library of Medicine (Bethesda, MD): Available: http://www.ncbi.nlm.nih.gov/omim/. Accessed 2012 Februrary 17

[33] Li R, Heydon K, Hammond ME, Grignon DJ, Roach M (2004). Ki-67 staining index predicts distant metastasis and survival in locally advanced prostate cancer treated with radiotherapy: an analysis of patients in radiation therapy oncology group protocol 86-10.. Clin Cancer Res.

[34] Bono AV, Celato N, Cova V, Salvadore M, Chinetti S (2002). Microvessel density in prostate carcinoma.. Prostate Cancer Prostatic Dis.

[35] de la Taille A, Katz AE, Bagiella E, Buttyan R, Sharir S (2000). Microvessel density as a predictor of PSA recurrence after radical prostatectomy. A comparison of CD34 and CD31.. Am J Clin Pathol.

[36] Moul JW (1999). Angiogenesis, p53, bcl-2 and Ki-67 in the progression of prostate cancer after radical prostatectomy.. Eur Urol.

[37] Bettencourt MC, Bauer JJ, Sesterhenn IA, Mostofi FK, McLeod DG (1996). Ki-67 expression is a prognostic marker of prostate cancer recurrence after radical prostatectomy.. J Urol.

[38] Bubendorf L, Sauter G, Moch H, Schmid HP, Gasser TC (1996). Ki67 labelling index: an independent predictor of progression in prostate cancer treated by radical prostatectomy.. J Pathol.

[39] Cohen RJ, Glezerson G, Haffejee Z (1992). Prostate-specific antigen and prostate-specific acid phosphatase in neuroendocrine cells of prostate cancer.. Arch Pathol Lab Med.

[40] Cohen RJ, Glezerson G, Haffejee Z, Afrika D (1990). Prostatic carcinoma: histological and immunohistological factors affecting prognosis.. Br J Urol.

[41] Bates RJ, Chapman CM, Prout GR, Lin CW (1982). Immunohistochemical identification of prostatic acid phosphatase: correlation of tumor grade with acid phosphatase distribution.. J Urol.

[42] Gunia S, Koch S, May M, Dietel M, Erbersdobler A (2009). Expression of prostatic acid phosphatase (PSAP) in transurethral resection specimens of the prostate is predictive of histopathologic tumor stage in subsequent radical prostatectomies.. Virchows Arch.

[43] Oracle website.. http://www.oracle.com/technetwork/java/index.html.

[44] Aperio (2008).

[45] Thevenaz P, Ruttimann UE, Unser M (1998). A pyramid approach to subpixel registration based on intensity.. IEEE Trans Image Process.

[46] TurboReg website.. http://bigwww.epfl.ch/thevenaz/turboreg/.

[47] Metropolis N, Ulam S (1949). The Monte Carlo method.. J Am Stat Assoc.

[48] Cooper L, Sertel O, Kong J, Lozanski G, Huang K (2009). Feature-based registration of histopathology images with different stains: an application for computerized follicular lymphoma prognosis.. Comput Methods Programs Biomed.

[49] Sun Y, Goodison S (2009). Optimizing molecular signatures for predicting prostate cancer recurrence.. Prostate.

[50] Bismar TA, Demichelis F, Riva A, Kim R, Varambally S (2006). Defining aggressive prostate cancer using a 12-gene model.. Neoplasia.

[51] Stephenson AJ, Smith A, Kattan MW, Satagopan J, Reuter VE (2005). Integration of gene expression profiling and clinical variables to predict prostate carcinoma recurrence after radical prostatectomy.. Cancer.

[52] Glinsky GV, Berezovska O, Glinskii AB (2005). Microarray analysis identifies a death-from-cancer signature predicting therapy failure in patients with multiple types of cancer.. J Clin Invest.

[53] Glinsky GV, Glinskii AB, Stephenson AJ, Hoffman RM, Gerald WL (2004). Gene expression profiling predicts clinical outcome of prostate cancer.. J Clin Invest.

[54] Febbo PG, Sellers WR (2003). Use of expression analysis to predict outcome after radical prostatectomy.. J Urol.

[55] Abd El-Rehim DM, Ball G, Pinder SE, Rakha E, Paish C (2005). High-throughput protein expression analysis using tissue microarray technology of a large well-characterised series identifies biologically distinct classes of breast cancer confirming recent cDNA expression analyses.. Int J Cancer.

[56] Makretsov NA, Huntsman DG, Nielsen TO, Yorida E, Peacock M (2004). Hierarchical clustering analysis of tissue microarray immunostaining data identifies prognostically significant groups of breast carcinoma.. Clin Cancer Res.

[57] Vendrell JA, Robertson KE, Ravel P, Bray SE, Bajard A (2008). A candidate molecular signature associated with tamoxifen failure in primary breast cancer.. Breast Cancer Res.

[58] Landemaine T, Jackson A, Bellahcene A, Rucci N, Sin S (2008). A six-gene signature predicting breast cancer lung metastasis.. Cancer Res.

[59] Charpin C, Secq V, Giusiano S, Carpentier S, Andrac L (2009). A signature predictive of disease outcome in breast carcinomas, identified by quantitative immunocytochemical assays.. Int J Cancer.

[60] Simone NL, Bonner RF, Gillespie JW, Emmert-Buck MR, Liotta LA (1998). Laser-capture microdissection: opening the microscopic frontier to molecular analysis.. Trends Genet.

[61] Dolled-Filhart M, Gustavson M, Camp RL, Rimm DL, Tonkinson JL (2010). Automated analysis of tissue microarrays.. Methods Mol Biol.

[62] Haedicke W, Popper HH, Buck CR, Zatloukal K (2003). Automated evaluation and normalization of immunohistochemistry on tissue microarrays with a DNA microarray scanner.. Biotechniques.

[63] Fritzsche FR, Bode PK, Moch H, Kristiansen G, Varga Z (2010). Determination of the Her-2/neu gene amplification status in cytologic breast cancer specimens using automated silver-enhanced in-situ hybridization (SISH).. Am J Surg Pathol.

[64] Laakso M, Tanner M, Isola J (2006). Dual-colour chromogenic in situ hybridization for testing of HER-2 oncogene amplification in archival breast tumours.. J Pathol.

[65] Hammond ME, Hayes DF, Dowsett M, Allred DC, Hagerty KL (2010). American Society of Clinical Oncology/College of American Pathologists guideline recommendations for immunohistochemical testing of estrogen and progesterone receptors in breast cancer (unabridged version).. Arch Pathol Lab Med.

[66] Ciocca DR, Elledge R (2000). Molecular markers for predicting response to tamoxifen in breast cancer patients.. Endocrine.

[67] Allred DC, Bustamante MA, Daniel CO, Gaskill HV, Cruz AB (1990). Immunocytochemical analysis of estrogen receptors in human breast carcinomas. Evaluation of 130 cases and review of the literature regarding concordance with biochemical assay and clinical relevance.. Arch Surg.

[68] Vance GH, Barry TS, Bloom KJ, Fitzgibbons PL, Hicks DG (2009). Genetic Heterogeneity in HER2 Testing in Breast Cancer Panel Summary and Guidelines.. Archives of Pathology & Laboratory Medicine.

[69] Lewis JT, Ketterling RP, Halling KC, Reynolds C, Jenkins RB (2005). Analysis of intratumoral heterogeneity and amplification status in breast carcinomas with equivocal (2+) HER-2 immunostaining.. American Journal of Clinical Pathology.

[70] Simon R, Nocito A, Hubscher T, Bucher C, Torhorst J (2001). Patterns of HER-2/neu amplification and overexpression in primary and metastatic breast cancer.. Journal of the National Cancer Institute.

[71] Lee CK, Sunkin SM, Kuan C, Thompson CL, Pathak S (2008). Quantitative methods for genome-scale analysis of in situ hybridization and correlation with microarray data.. Genome Biol.

[72] Lein ES, Hawrylycz MJ, Ao N, Ayres M, Bensinger A (2007). Genome-wide atlas of gene expression in the adult mouse brain.. Nature.

